# DISCOHAT: An Acronym to Describe the Spectrum of Symptoms Related to Bilateral Vestibulopathy

**DOI:** 10.3389/fneur.2021.771650

**Published:** 2021-11-12

**Authors:** Sophie Paredis, Lisa van Stiphout, Eva Remmen, Michael Strupp, Marie-Cecile Gerards, Herman Kingma, Vincent Van Rompaey, Angelica-Perez Fornos, Nils Guinand, Raymond van de Berg

**Affiliations:** ^1^Maastricht University Medical Centre, Maastricht, Netherlands; ^2^Department of Neurology and Neurological Intensive Care, Munich Hospital, Munich, Germany; ^3^Antwerp University Hospital, Antwerp, Belgium; ^4^Geneva University Hospitals (HUG), Geneva, Switzerland; ^5^Centre Médical Universitaire, Université de Genève, Geneva, Switzerland

**Keywords:** history taking, vestibular disorders, vestibulopathy, vestibular hypofunction, vestibular areflexia

## Abstract

**Objective:** To assess the prevalence of each symptom listed in the acronym DISCOHAT (worsening of symptoms in Darkness and/or uneven ground, Imbalance, Supermarket effect, Cognitive complaints, Oscillopsia, Head movements worsen symptoms, Autonomic complaints, and Tiredness) in patients with bilateral vestibulopathy (BVP), compared to patients with unilateral vestibulopathy (UVP).

**Methods:** A descriptive case-control study was performed on BVP and UVP patients who were evaluated for their vestibular symptoms by two of the authors (RvdB, MCG) at a tertiary referral center, between 2017 and 2020. During history taking, the presence of each DISCOHAT symptom was checked and included in the electronic health record. Presence of a symptom was categorized into: “present,” “not present,” and “missing.”

**Results:** Sixty-six BVP patients and 144 UVP patients were included in this study. Prevalence of single DISCOHAT symptoms varied from 52 to 92% in BVP patients and 18–75% in UVP patients. Patients with BVP reported “worsening of symptoms in darkness,” “imbalance,” “oscillopsia,” and “worsening of symptoms with fast head movements” significantly more than UVP patients (*p* ≤ 0.004).

**Conclusion:** The DISCOHAT acronym is able to capture a wide spectrum of symptoms related to vestibulopathy, while it is easy and quickly to use in clinic. Application of this acronym might facilitate a more thorough and uniform assessment of bilateral vestibulopathy, within and between vestibular clinics worldwide.

## Introduction

Bilateral vestibulopathy (BVP) is a chronic condition in which the vestibular function is reduced or absent due to hypofunction of both vestibular organs, the vestibular nerves and/or central neural structures (1, 2). The main symptoms are imbalance/unsteadiness and oscillopsia (the illusory movement of the environment), due to the loss of, respectively, vestibulo-spinal and vestibulo-ocular reflexes. These symptoms can have a great impact on quality of life (1–3). As much as 44% of patients experience BVP as a severe handicap while 41% perceive it as a moderate handicap (2, 4).

The Classification Committee of the Bárány Society described the diagnostic criteria for BVP, using a combination of symptoms, signs and laboratory testing results (1). The diagnosis BVP can be challenging and it is therefore often missed or misdiagnosed (5). One of the main challenges of diagnosing BVP, is the fact that disorders resulting in BVP can have different clinical presentations: patients can present with and without vertigo (6), and while imbalance and oscillopsia are the most well-known symptoms of BVP (7), a wide spectrum of other symptoms can be present in BVP patients (8). For example, visually induced dizziness (previously called “the supermarket effect”) involving dizziness triggered by visual stimuli like fast moving objects and certain patterns, is often reported (9). Patients can also have cognitive complaints such as difficulties when trying to perform dual tasks or when navigating (9–12), and symptoms often worsen when they perform fast head movements. Furthermore, symptoms like tiredness, dizziness upon standing up, vertigo attacks, light headedness, tinnitus, neck pain, nausea, headache, a restless mind and problems with sleeping (8, 11, 13) can be present in BVP patients. All these symptoms might reflect different mechanisms, like loss of VOR (e.g., oscillopsia), visuo-vestibular mismatch (e.g., visually induced dizziness) (14) or vestibular-autonomic interactions (e.g., dizziness upon standing up) (15). Some symptoms can also share their etiology with the vestibulopathy (e.g., hearing loss and tinnitus in Menière's disease).

Although imbalance/unsteadiness and/or oscillopsia are the symptoms necessary to diagnose BVP, structured reporting of the additional symptoms might be valuable for treatment of BVP in clinical and/or research settings. The prognosis of BVP is poor (16), but vestibular rehabilitation is possible (17) and therapeutic devices like vibrotactile feedback systems (18), and vestibular implants (19–22) are emerging. A thorough inventory of the spectrum of symptoms related to BVP, might therefore improve the individual rehabilitation process by e.g., facilitating goal setting during rehabilitation (23). Next to this, a good insight in the spectrum of symptoms could improve the development of patient reported outcome measures, necessary to reliably investigate new therapeutic devices (24).

A BVP-specific questionnaire is unfortunately not (yet) available. Based on a previous study (6), this study therefore proposes an acronym that is able to easily and quickly capture a spectrum of symptoms in clinic, directly related to vestibulopathy: DISCOHAT. The acronym includes:

Darkness: worsening of symptoms in darkness and/or on uneven ground (1)
Imbalance: unsteadiness when walking or standing (1)
Supermarket effect: visually induced dizziness: intolerance to busy visual environments
Cognitive complaints: difficulties with memory, concentrating, dual-tasking, navigation, etc.
Oscillopsia: illusory movement of the environment due to failure of gaze stabilization
Head movements: worsening of symptoms with fast head movements (e.g., look left and right to cross the street)
Autonomic complaints: signs of dysautonomia, like dizziness upon standing up
Tiredness: increase of tiredness due to symptoms related to vestibulopathy
DISCOHAT might facilitate a more thorough and uniform assessment of BVP patients, within and between vestibular clinics worldwide.

The objective of this study was to assess the prevalence of each symptom listed in the acronym DISCOHAT, in patients with bilateral vestibulopathy. Since many of these symptoms reflect vestibulopathy in general, prevalences were compared to patients with unilateral vestibulopathy (UVP). It was hypothesized that some symptoms like worsening of symptoms in darkness, imbalance and oscillopsia would be more prevalent in BVP patients due to the difference in vestibular function.

## Methods

### Patient Inclusion

A descriptive case-control study was performed on all patients with BVP and UVP who were evaluated for their vestibular symptoms by two of the authors (RvdB, MCG) at the vestibular department of Maastricht University Medical Center between 01-01-2017 and 01-04-2020. Bilateral vestibulopathy was defined according to the diagnostic criteria of the Classification Committee of the Bárány Society, which included a history of imbalance/unsteadiness possibly combined with oscillopsia, and a bilaterally reduced or absent vestibulo-ocular reflex (VOR) function as documented by video Head impulse testing (VOR gain < 0.6) and/or rotatory chair testing (VOR gain < 0.1) and/or caloric testing (sum of bithermal maximum peak slow phase velocities of nystagmus <6°/s on each side) (1). Unilateral vestibulopathy (UVP) was defined as the presence of vestibular symptoms (e.g., dizziness and imbalance) combined with the presence of a caloric asymmetry of at least 25%, measured using the Jonkees formula (25). Patients were excluded in case they were <18 years old at the time of inclusion, when they were included in the previous BVP study which was used as the basis for the development of the DISCOHAT acronym (6) (exclusion facilitated external validation).

### Data Collection: History Taking and DISCOHAT

The presence of each DISCOHAT symptom was evaluated by two of the authors during routine clinical history taking of vestibular patients. These two authors were instructed and trained to use the acronym DISCOHAT in patients with BVP and UVP. During history taking, the smartphrase “vertigodiscohat” could be used in the electronic health records (SAP ISH Cerner 2009), which provided the DISCOHAT symptoms. In case patients suffered from an episodic vestibular syndrome (e.g., Menière's disease) combined with chronic complaints of BVP or UVP, the presence of DISCOHAT symptoms was explicitly only evaluated regarding the chronic complaints, not the attacks. The presence of a symptom was not only checked by asking whether it was “yes” or “not” present: if necessary, patients were asked to further clarify their answer. After clarification, the authors decided whether a symptom was present or not (e.g., oscillopsia might initially seem present when a patients indicates a blurred vision while moving, but this could change after further elaboration on the symptom “blurred vision while moving”). The presence or absence of a symptom was included in the electronic health record.

### Vestibular Testing

All patients underwent vestibular testing at the vestibular department of Maastricht University Medical Center+, which was performed by trained laboratory technicians. The testing paradigms were previously described in detail (13, 23). To summarize, caloric tests (Variotherm Plus device, Lenzkirch, Germany) were performed with warm (44°C) and cold (30°C) water. For each irrigation, 300 ml water was irrigated during 30 s. Torsion swing tests (Ekida GmbH, Buggingen, Germany) were performed with sinusoidal rotation (0.1 Hz) with a peak velocity of 60°/s. The video Head Impulse Test was performed using the Otometrics system (Otometrics, Taastrup, Denmark). Head impulses comprised fast (peak velocity > 150°), unpredictable, low-amplitude (±20°) head movements in the horizontal plane.

### Data Analysis

Patient data was included in the analysis when at least 7 out of 8 DISCOHAT symptoms were evaluated during clinical history taking. In case 7 out of 8 symptoms were obtained, the remaining symptom was considered as missing data. As this was the first exploratory study using the acronym DISCOHAT, presence of a symptom was categorized into: “present,” “not present,” and “missing.” Only symptoms present at the time of consultation were considered “present.” No grading system to assess the severity of the symptoms was used. Disease duration was calculated in years, starting from the onset of the first complaints.

Descriptive statistics were applied. IBM SPSS Statistics 26 (IBM) and Microsoft Excel (Microsoft Office 365) were used for statistical analysis. The chi-squared test was applied to test for differences and correlations in nominal values and the independent *T*-test was used to test for differences in numeric values. Missing data was not included in this analysis. The significance level was set at 0.05 and Bonferoni correction was applied to correct for multiple testing.

### Ethical Considerations

This study was performed in accordance with the guidelines outlined by Dutch legislation. According to the Medical Research involving Human Subjects Act (WMO) ethical approval was not required due to the retrospective nature and anonymization of this study.

## Results

### Patient Characteristics

**Figure 1:**
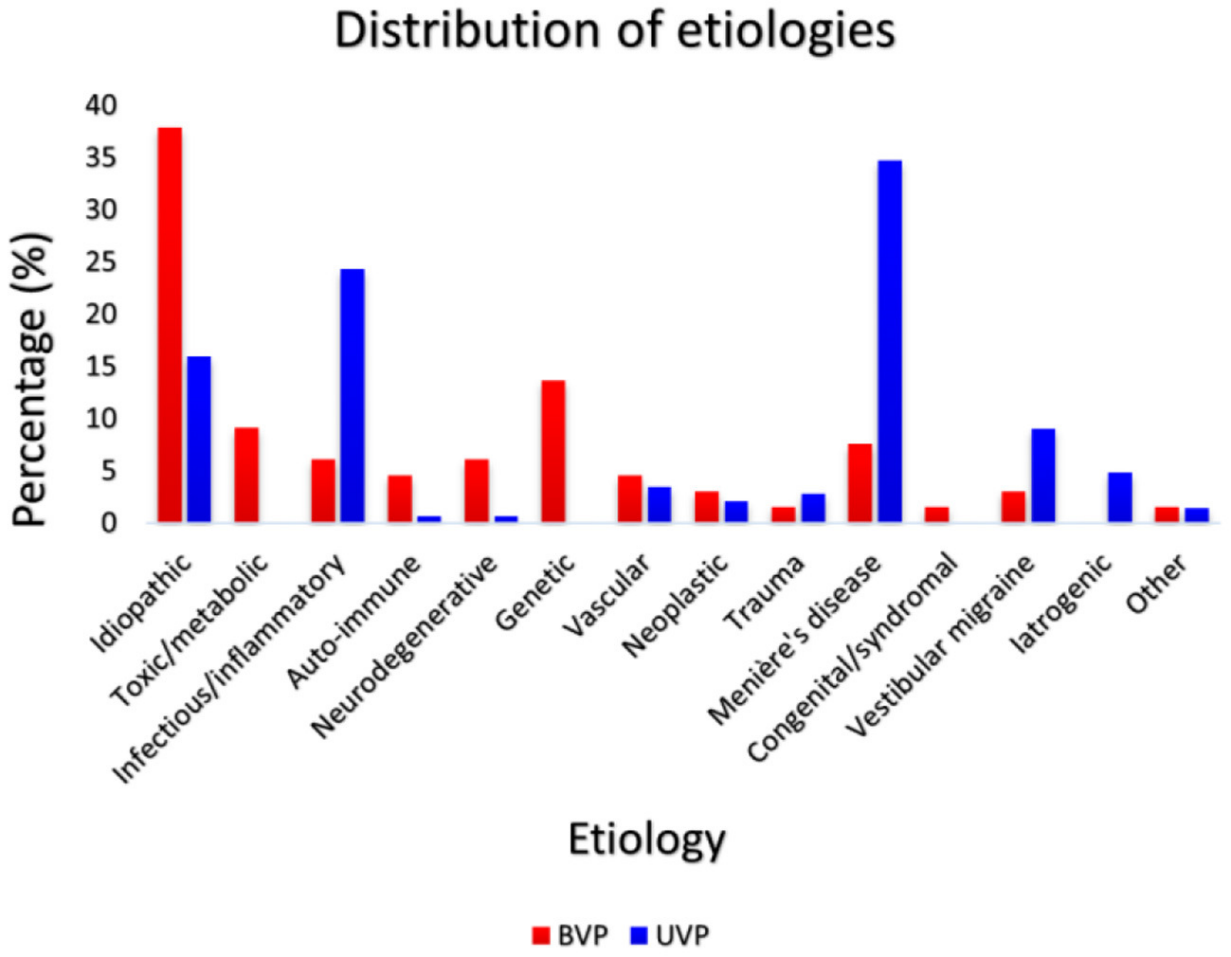
The distribution of etiologies within and between the BVP (*N* = 66) and UVP groups (*N* = 144). Red bars represent the percentage of each etiology in the BVP group and dark blue bars represent the percentage of each etiology in the UVP group.

At least seven out of eight DISCOHAT symptoms were evaluated in 66 BVP patients and in 144 UVP patients. Fifty-three percent of BVP patients were male and 40% of UVP patients were male. Mean age of BVP patients was 58 years old (range 23–85 years) and mean age of UVP patients was 59 years old (range 18–84 years). Both gender and age did not significantly differ between groups (*p* = 0.068 and *p* = 0.582, respectively). Mean disease duration was significantly different between BVP patients (10 years; range 1–40 years; SD 10 years) and UVP patients (7 years; range 1–42 years, SD 8 years) for UVP patients, *p* = 0.021. Etiologies differed between the BVP and UVP groups. Figure 1 presents the distribution of etiologies (13) within and between the BVP and UVP groups. It can be observed that a genetic cause (mainly DFNA9) was the most common etiology in the BVP group, while this involved Meniere's disease in the UVP group. A definite diagnosis was made in 23% of BVP patients and 53% of UVP patients, and a probable diagnosis was made in 39% of BVP patients and 31% of UVP patients. Thirty-nine percent of BVP diagnoses and 16% of UVP diagnoses remained idiopathic.

### Presence of DISCOHAT Symptoms in BVP and UVP Patients

**Figure 2:**
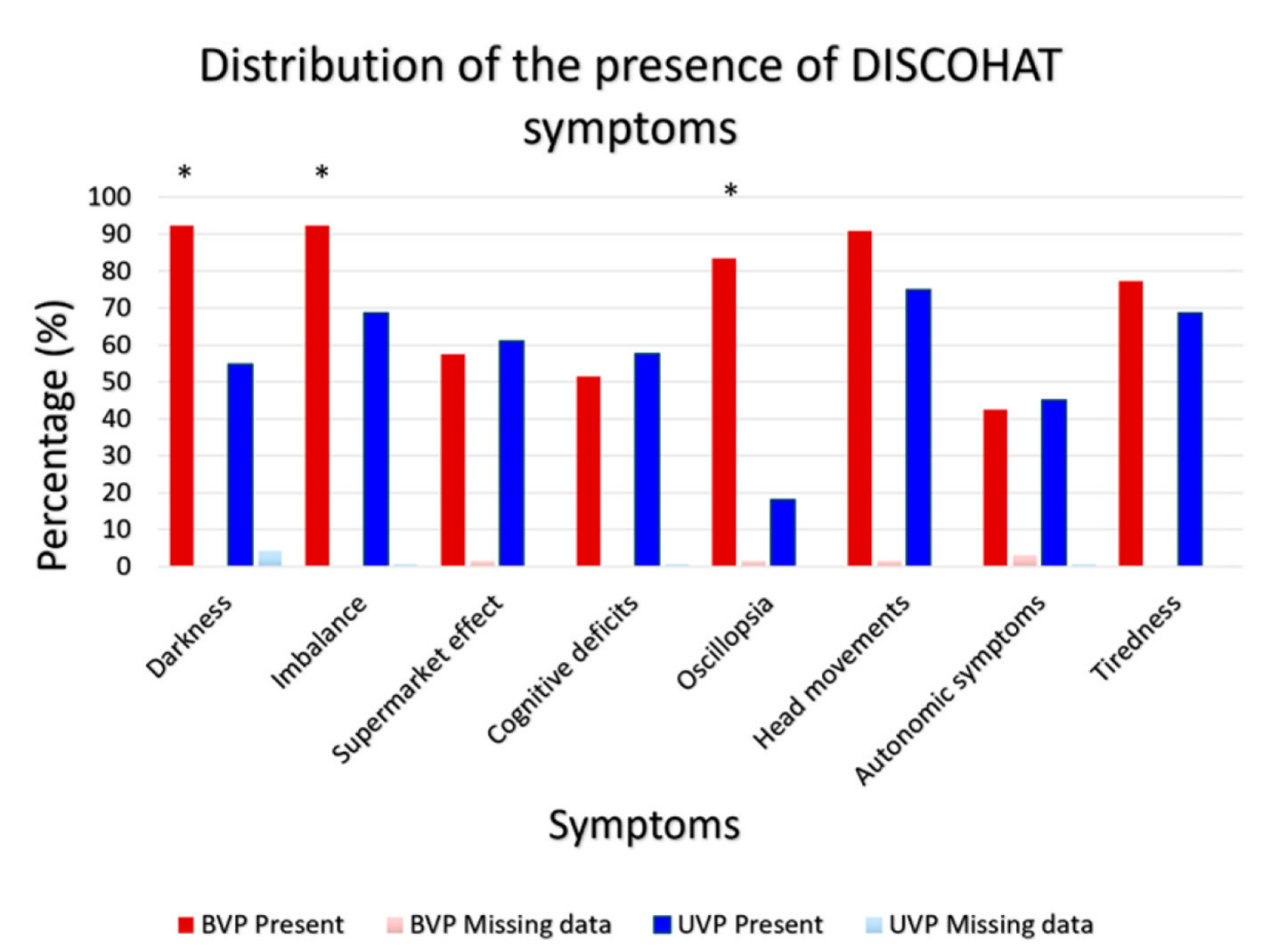
Distribution of the presence of DISCOHAT symptoms in the BVP group (*N* = 66) and UVP group (*N* = 144). Red bars illustrate the presence (%) of each symptom within the BVP group and the dark blue bars illustrate the presence (%) of each symptom within the UVP group. An asterisk indicates a statistically significant difference regarding the presence (yes/no) of that specific symptom, between the BVP and UVP groups.

Figure 2 illustrates the presence of the eight DISCOHAT symptoms in the BVP and UVP groups. Both BVP as well as UVP patients often reported presence of DISCOHAT symptoms. It can be observed that each DISCOHAT symptom was present in at least 52–92% of BVP patients and 18–75% of UVP patients. In BVP patients, “worsening of symptoms in darkness,” “imbalance,” and “worsening of symptoms with fast head movements” were most present, while autonomic complaints were least reported. UVP patients reported most often the presence of “worsening of symptoms with fast head movements,” while “oscillopsia” was least reported. Between groups, BVP patients reported the presence of “worsening of symptoms in darkness” “imbalance,” “oscillopsia,” and “worsening of symptoms significantly more with fast head movements” than UVP patients (*p* ≤ 0.004). The other symptoms did not show any significant differences between groups regarding their presence.

The time to obtain DISCOHAT symptoms was not monitored. However, the two authors who performed history taking, indicated an average time to reliably obtain DISCOHAT symptoms of ~1–2 min.

## Discussion

This study evaluated the presence of DISCOHAT symptoms in BVP patients compared to UVP patients. It was shown that all DISCOHAT symptoms were frequently reported in both groups, except oscillopsia in UVP patients (18%). BVP patients reported “worsening of symptoms in darkness,” “imbalance,” “oscillopsia,” and “worsening of symptoms with head movements” significantly more than UVP patients. This is the first time that this spectrum of symptoms was assessed in BVP and UVP patients, using a structured approach.

The advantage of the DISCOHAT acronym is the fact that it structurally captures a spectrum of symptoms related to vestibulopathy, while it is easy and quickly to use in clinic. This could facilitate uniform documentation of these symptoms within and between clinics world-wide. Such a structured documentation might be valuable during rehabilitation (23) and when developing therapeutic devices and patient reported outcome measures (1, 20, 23, 25). The DISCOHAT acronym is complementary to currently used paradigms in history taking of vestibular patients, like focusing on “timing and triggers” (26) and the “SO STONED” paradigm (27). After all, these two paradigms mainly describe how to perform history taking in general, while DISCOHAT describes specifically what can be asked related to vestibulopathy. Regarding scoring the presence of DISCOHAT symptoms, the authors strongly advise to motivate patients to clarify their answers to the DISCOHAT questions. After all, some symptoms (especially oscillopsia) might be difficult to identify with only one or two questions. Where in doubt as to whether the patient understood the concept correctly, patients were asked to describe the symptom in specific situations. For example, a patient who reports “blurred vision while walking,” might additionally report “bouncing of the horizon while driving on a bumpy road.” This increases the chance of having oscillopsia. In contrast, if the patient would have reported that the blurred vision is also present without any head movements and that it can be corrected by wearing eye glasses, this would make oscillopsia less likely. In this study not all symptoms which appeared to be present at first sight, seemed to be truly present after clarification, and vice versa.

Most DISCOHAT symptoms were frequently reported by both BVP and UVP patients. In a previous study using semi-structured qualitative interviews, these symptoms were already identified as being frequently present in BVP patients (6). Since the DISCOHAT acronym was not used in the previous study, this current study explicitly used a different group of BVP patients to externally validate the DISCOHAT acronym. The reported prevalences of symptoms indicate that the DISCOHAT acronym is able to capture a wide spectrum of relevant BVP symptoms, beyond only “imbalance” and “oscillopsia.” As expected, BVP patients reported the presence of specific symptoms like imbalance, oscillopsia, worsening of symptoms in darkness and worsening of symptoms with fast head movements significantly more. This might partially reflect the difference in vestibular function compared to UVP patients, as well as the diagnostic criteria for BVP. After all, three out of these four symptoms are also part of the diagnostic criteria for BVP. Nevertheless, UVP patients also often reported the presence of DISCOHAT symptoms. It could therefore be hypothesized that DISCOHAT symptoms are not only a reflection of bilateral vestibulopathy, but also of vestibulopathy in general (28–30). For example, in this population of UVP patients, 69% of patients reported imbalance and 18% reported oscillopsia. This is congruent with previous literature which illustrated that UVP patients can still experience multiple symptoms including postural instability and visual blurring with head movement (31–33).

Regarding visually induced dizziness (the “supermarket effect”) it can be sometimes challenging to determine whether this symptoms results from the vestibulopathy or from co-morbid Persistent Postural-Perceptual Dizziness. Therefore in case visually induced dizziness is reported, it is advised to actively investigate the presence of co-morbid Persistent Postural-Perceptual Dizziness (34).

It should be noted that the group of UVP patients were recruited at a tertiary referral center, and prevalences most likely reflect a tertiary referral center population: a selection bias cannot be ruled out (e.g., patients with multiple and/or severe symptoms that require a second opinion). Nevertheless, these results again demonstrate that not all UVP patients perfectly compensate (21). Furthermore, mean disease duration for BVP and UVP patients was 10 and 7 years, respectively. Since vestibular compensation for static symptoms can take up to only 1 year and vestibular compensation for dynamic symptoms often remains poor (35), it is unlikely that the significantly shorter disease duration of UVP patients might have compromised comparison of results between BVP and UVP patients.

The presence of some symptoms (e.g., cognitive deficits and tiredness) did not significantly differ between UVP and BVP patients. This does not imply an equal severity of symptoms between BVP and UVP patients. After all, the presence of DISCOHAT symptoms (yes or no) was scored in this study, but the severity was not graded. Further development of assessment tools regarding the wide spectrum of vestibulopathy symptoms, should therefore include also grading of severity (e.g., using a Likert scale) (36). Currently, the authors of this study are working on such a questionnaire tailored to the symptoms of bilateral vestibulopathy, in accordance with the COSMIN methodology (37).

Finally, BVP can often result in physical, cognitive and emotional complaints (6). DISCOHAT comprises mainly the physical and cognitive domain, but the emotional domain might be underrepresented. Although the autonomic nervous system (the “A” in DISCOHAT) and emotions are closely correlated (38), it could be proposed to more explicitly include emotional complaints, by adding the “E”: DISCOHEAT.

### Limitations

Three main limitations were identified in this study. First, no age-matched healthy control group was used. Therefore, it cannot be ruled out that some DISCOHAT symptoms are also to a certain extent present in the healthy population. However, four out of eight DISCOHAT symptoms were significantly more present in BVP patients than in UVP patients. This illustrates that the presence of these symptoms seem to be related to the lack of vestibular function. Furthermore, the remaining symptoms (visually induced dizziness, cognitive and autonomic complaints, and tiredness) can also be relevant reasons for medical consultation, especially in case of disabling severity (39, 40). It would therefore be valuable, as stated above, to include grading of symptoms and not only presence. Secondly, not all eight DISCOHAT symptoms were evaluated in every patient seen by the two authors. This might give the impression of a selection bias. However, main reason to not include the whole DISCOHAT acronym during history taking, was lack of time during consultation (although evaluation even took approximately only 1–2 min). A selection bias was avoided as much as possible by the two authors. Thirdly, etiologies differed between BVP and UVP patients. Episodic vestibular syndromes (e.g., Meniere's disease) were more present in the UVP group. Since vertigo attacks can present with different symptoms than BVP or UVP, the two authors explicitly only evaluated the presence of DISCOHAT symptoms regarding the chronic complaints, not the attacks. This facilitated, as much as possible, evaluation of symptoms most likely related to BVP and UVP.

## Conclusion

The DISCOHAT acronym is able to capture a wide spectrum of symptoms related to vestibulopathy, while it is easy and quickly to use in clinic. Application of this acronym might facilitate a more thorough and uniform assessment of bilateral vestibulopathy, within and between vestibular clinics worldwide.

## Data Availability Statement

The original contributions presented in the study are included in the article/supplementary material, further inquiries can be directed to the corresponding authors.

## Ethics Statement

Ethical review and approval was not required for the study on human participants in accordance with the local legislation and institutional requirements. The patients/participants provided their written informed consent to participate in this study.

## Author Contributions

RB and LS contributed to conception and design of the study. ER, LS, and SP organized the database. ER and LS performed the statistical analysis. RB and M-CG collected the data in clinical practice. RB and MS read and approved the submitted version. All authors contributed to manuscript revision.

## Conflict of Interest

The authors declare that the research was conducted in the absence of any commercial or financial relationships that could be construed as a potential conflict of interest.

## Publisher's Note

All claims expressed in this article are solely those of the authors and do not necessarily represent those of their affiliated organizations, or those of the publisher, the editors and the reviewers. Any product that may be evaluated in this article, or claim that may be made by its manufacturer, is not guaranteed or endorsed by the publisher.
